# Uniporter substrate binding and transport: reformulating mechanistic questions

**DOI:** 10.1007/s41048-016-0030-7

**Published:** 2016-10-27

**Authors:** Xuejun C. Zhang, Lei Han

**Affiliations:** National Laboratory of Biomacromolecules, CAS Center for Excellence in Biomacromolecules, Institute of Biophysics, Chinese Academy of Sciences, Beijing, 100101 China

**Keywords:** Uniporter, GLUT1, Energy coupling, Kinetics

## Abstract

Transporters are involved in material transport, signaling, and energy input in all living cells. One of the fundamental questions about transporters is concerned with the precise role of their substrate in driving the transport process. This is particularly important for uniporters, which must utilize the chemical potential of substrate as the only energy source driving the transport. Thus, uniporters present an excellent model for the understanding of how the difference in substrate concentration across the membrane is used as a driving force. Local conformational changes induced by substrate binding are widely considered as the main mechanism to drive the functional cycle of a transporter; in addition, reducing the energy barrier of the transition state has also been proposed to drive the transporter. However, both points of view require modification to allow consolidation with fundamental thermodynamic principles. Here, we discuss the relationship between thermodynamics and kinetics of uniporters. Substrate binding-induced reduction of the transition-state energy barrier accelerates the transport process in kinetic terms, while the chemical potential of the substrate drives the process thermodynamically.

## Uniporters

Uniporters are integral membrane proteins that transport substrates across the cellular membrane by solely using the chemical potential of the substrates as their driving force (Naftalin and De Felice 2012). Depending on the direction of substrate concentration gradient, a uniporter can transport its substrate in either influx or efflux directions, yet the influx and efflux transports are usually of distinct kinetics. One of the most extensively studied eukaryotic uniporters is the glucose transporter, GLUT1 (Carruthers et al. 2009; Deng and Yan 2016). It was first purified and characterized in 1970s (Kasahara and Hinkle 1977), with its crystal structure reported only recently (Deng et al. 2014). Like other uniporters, GLUT1 can transport glucose in either direction. On the basis of these findings, a commonly asked question is how substrate binding drives this transport. In our opinion, this question is fundamentally flawed, as it does not take into consideration the thermodynamics of transport.

**Fig. 1:**
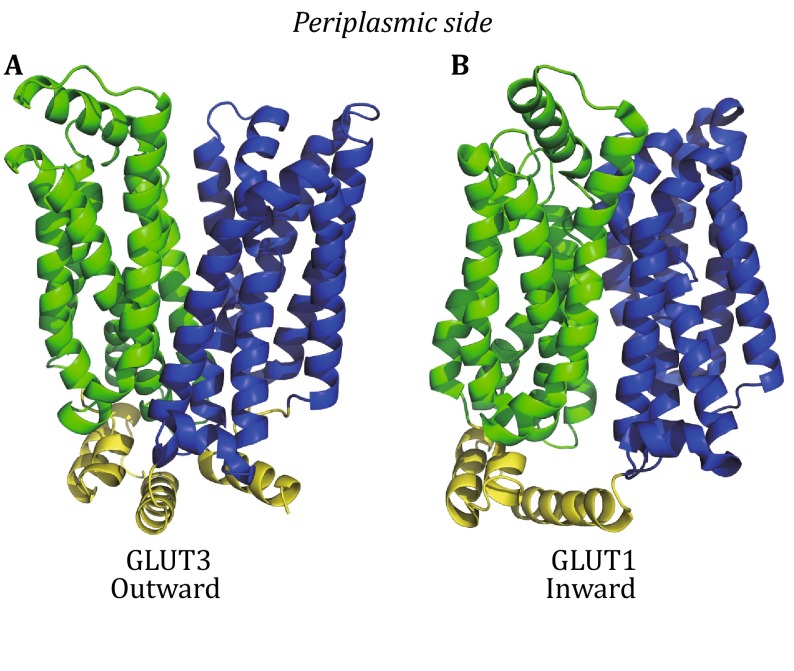
Crystal structures of representative MFS uniporters. **A** GLUT3 structure in the outward-facing state (PDB ID: 4ZWC). **B** GLUT1 structure in the inward-facing state (PDB ID: 4PYP). The N and C domains are colored *green* and *blue*, respectively, in both the structures, with the intracellular domain colored *yellow*

In 1960s, Jardetzky proposed a general alternating-access model for transporters, including uniporters (Jardetzky 1966), the first attempt to hypothesize on the connection between thermodynamics and the structure of a transporter. This model assumes three characteristic features: (1) a transporter must contain a cavity in its interior that is sufficiently large to accommodate the substrate; (2) it must be able to assume two different conformations to allow for the molecular cavity to be open to one side of the membrane in one conformation and to the opposite side in the other; and (3) it must contain a binding site for substrates in the cavity, the substrate affinity of which may be different in the two conformations. In 2003, crystal structures of two transporters from the major facilitator superfamily (MFS), LacY and GlpT, were reported in the inward-facing conformation (C_in_); and in 2010, the structure of FucP from the same superfamily was reported in the outward-facing conformation (C_out_) (Abramson et al. 2003; Huang et al. 2003; Dang et al. 2010). More recently, crystal structures of GLUT uniporters from the MFS family have also been reported in both the C_in_ and C_out_ states (Deng et al. 2014; Deng et al. 2015; Nomura et al. 2015) (Fig. 1). These structural studies illustrate the impressive accuracy of the alternating-access model, which is specifically termed as a “rocker-switch” model for MFS transporters. From a theoretical point of view, the Jardetzky model is a typical example of the so-called two-state model in physics, which has found broad applications in biology (Phillips et al. 2009). While there are claims that a two-state model is too simple to describe the complex properties of uniporters, such as *trans*-acceleration and asymmetric transport (Carruthers et al. 2009; Naftalin and De Felice 2012), we believe that this model can provide mechanistic explanations to these seemingly complicated phenomena. There are more complicated cases in which the transport process of a uniporter may deviate from the two-state model, for example being allosterically regulated or containing loops in addition to the major reaction cycle. However, in most cases, the two-state model would be a good starting point to dissect the transport mechanism.

## Two-state model for a transport cycle

**Fig. 2:**
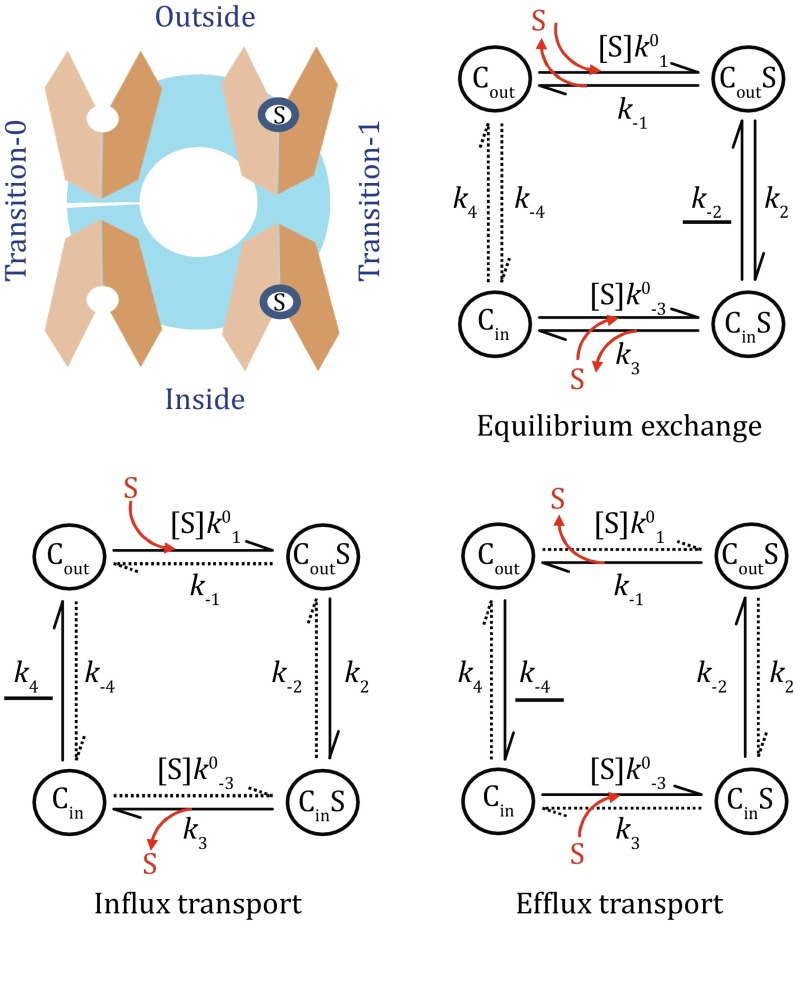
Two-state four-step model. The *top-left panel* is a schematic presentation of the two-state model, and the remaining are its King–Altman diagrams in different types of transport. In each type of transport, dominant paths are shown in *solid lines*, and the rate-limiting step (for GLUT1) is *underlined*

In the two-state model, each of the two conformations, C_in_ and C_out_, of a given uniporter may have two sub-states, i.e., being either occupied or unoccupied by the substrate. Thus, a transport cycle of the uniporter can be presented with a two-state, four-step King–Altman plot (Fig. 2). In thermodynamic terms, such a cycle can be described by just three independent parameters, which can be chosen from *f*(0), *f*(∞), [S]_in_/*K*_d,in_, [S]_out_/*K*_d,out_, ∆*G*_E_, or ∆*G*_D_ (Zhang et al. 2015). These parameters are introduced in the next three paragraphs, followed by examples of GLUT transporters.

The partition function *f*([S]) (≡([C_out_] + [C_out_S])/([C_in_] + [C_in_S])) describes the ratio of C_out_ to C_in_ as a function of substrate concentration, and *f*(0) and *f*(∞) are the corresponding values at zero and saturated substrate concentration, respectively (Zhang et al. 2015). The curve of *f*([S]) vs [S] can be measured experimentally, using techniques such as single-molecule Förster resonance energy transfer (smFRET) or double electron–electron resonance (DEER) (Smirnova et al. 2007; Akyuz et al. 2015; Heng et al. 2015).

Among the above-mentioned parameters, those favored by biochemists are perhaps the dissociation constants *K*_d,in_ and *K*_d,out_ in the C_in_ and C_out_ states, respectively (see Appendix 1). These parameters can be calculated from *f*([S]), provided that *f*(0) ≠ *f*(∞) (Zhang et al. 2015). Most substrate-binding assays used in studying transporters, such as surface plasma resonance (SPR), isothermal titration calorimetry (ITC), or scintillation proximity assay (SPA), provide an apparent dissociation constant, *K*_d,app_, which is neither *K*_d,in_ nor *K*_d,out_, but is a weighted average value of both (Zhang et al. 2015). In addition, a possibility that the “transition” state can be stabilized by substrate in the in vitro assay may further complicate interpretations of the results from such a *K*_d_ measurement.

**Fig. 3:**
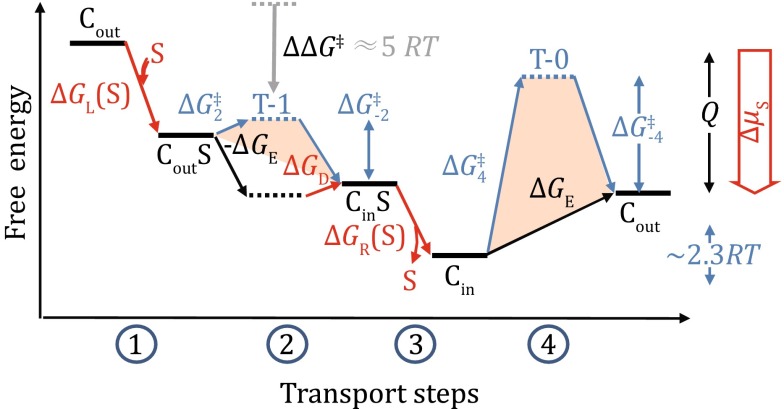
Schematics of the free-energy landscape of influx transport by GLUT1. A free-energy landscape plot describes the thermodynamic relationship between different states. The plot must satisfy the First and Second Laws of thermodynamics. Horizontal lines represent states. Tilted lines represent transitions between states. *Red arrows* are associated with the chemical potential of the substrate. Subscripts “L,” “R,” “D,” and “E” stand for energy terms associated with loading, releasing, differential binding, and empty carrier, respectively. The starting and ending states are identical, only being differed by the release of heat (*Q*) during one transport cycle. Experimental raw data from Lowe and Walmsley (1986) are reflected in the relative scales of the free-energy terms, but derived values of energy barriers of transition-1 (T-1) and transition-0 (T-0) are significantly reduced in the current plot (see Appendix 5). Note that for each and every ten-fold change in either population (such as life time or concentration) or kinetic rate, the corresponding free-energy change is 2.3 *RT* (i.e., *RT*ln(10)). In addition, since ∆*G*_D_ ≈ 0, for the substrate binding-induced reductions of the energy barrier ∆∆*G*_OI_^‡^ ≈ ∆∆*G*_IO_^‡^ (denoted as ∆∆*G* ^‡^). Assuming that a hydrogen bond contributes 2 *RT* (~5 kJ/mol) free energy, the 5 *RT* reduction in ∆*G* ^‡^ is equivalent to 2–3 hydrogen bonds

Free-energy terms ∆*G*_E_ and ∆*G*_D_ are the most important parameters in the two-state physics model (Phillips et al. 2009). ∆*G*_E_ 1 is the free-energy difference between C_in_ and C_out_ in the absence of the substrate. The corresponding conformational change is referred to as transition-0 (Figs. 2, 3). ∆*G*_E_ (≡−*RT*ln(*f*(0))) is directly related to the concentration ratio of the two conformations at zero substrate concentration, and a negative value would indicate that C_out_ represents a more stable state than C_in_. Furthermore, the differential binding energy, ∆*G*_D_, is defined as *RT*ln(*K*_d,in_/*K*_d,out_). On the one hand, ∆*G*_D_ is an intrinsic property of the transporter in a sense that its value is independent of the substrate concentration. The concept of ∆*G*_D_ has been implied in the Jardetzky’s original model. On the other hand, ∆*G*_D_ can be considered as part of the chemical potential (∆*µ*_S_) of the substrate, and the value of ∆*G*_D_ determines whether ∆*µ*_S_ contributes positively or negatively to the driving force for the substrate-carrying conformational change, which is referred to as transition-1. Furthermore, ∆*µ*_S_ (≡*RT*ln([S]_in_/[S]_out_), for influx transport) can be divided into three terms, namely (1) free energy of loading (L), ∆*G*_L_ (≡*RT*ln(*K*_d,out_/[S]_out_)); (2) free energy of releasing (R), ∆*G*_R_ (≡*RT*ln([S]_in_/*K*_d,in_)); and (3) the differential binding energy ∆*G*_D_ (Zhang et al. 2015). For a given ∆*µ*_S_ determined by the experimental condition, a favorable change in ∆*G*_D_ (e.g., by a mutation in the transporter) is necessarily accompanied by unfavorable changes in ∆*G*_L_ and/or ∆*G*_R_, in terms of facilitating the transport process. In addition, the combined term ∆*G*_D_ − ∆*G*_E_ (i.e., −*RT*ln(*f*(∞))) is the free-energy change associated with the substrate-carrying conformational change, transition-1. Thus, in principle, all of the above thermodynamic parameters can be calculated solely from values of the partition function *f*([S]) measured at three or more substrate concentrations. These parameters are sufficient to describe the thermodynamic cycle of a two-state model, which may serve as the basis for more sophisticated mathematic models for transporters.

Based on previously reported data at 0 °C, ∆*G*_E_ and ∆*G*_D_ of human GLUT1 are estimated to be +2.8 *RT* and +0.2 *RT*, respectively (Lowe and Walmsley 1986). The small value of ∆*G*_D_ suggests that *K*_d,in_ and *K*_d,out_ are nearly identical and that differential binding energy contributes almost zero to the driving force for GLUT1. Thus, what actually drives the transport process in this case can only originate from the ∆*G*_L_ and ∆*G*_R_ terms, by favoring the forward movement and/or preventing the backward movement (Zhang and Han 2016). In addition, in both the absence and presence of substrate, GLUT1 stays predominantly in the C_in_ state (with *f*(0) = 0.06 and *f*(∞) = 0.07), which is in agreement with the above-mentioned positive value of ∆*G*_E_. A recently reported 1.5-Å crystal structure of GLUT3 (PDB ID: 4ZW9) (Deng et al. 2015) revealed that a substrate glucose molecule forms multiple hydrogen bonds with amino acid residues from the central cavity of the transporter, and the binding site is relatively narrow. The hydrogen bonds (i.e., the enthalpy term in ∆*G*_L_ or ∆*G*_R_) contribute favorably to both substrate affinity and selectivity. However, while the narrow binding site contributes positively to the substrate selectivity, it negatively affects the affinity because of a decrease in entropy (see Appendix 1). Then, how these thermodynamic parameters are related to the kinetic properties of a uniporter remains to be discussed.

## Kinetics

Apart from considering thermodynamic parameters, understanding the precise mechanisms of substrate transport of a uniporter requires further kinetic information, i.e., the parameters *k*_1_^0^, *k*_−1_, and so on as shown in the King–Altman plot (Fig. 2). However, not all of these kinetic parameters act independently from each other; on the contrary, they are related via thermodynamic parameters (e.g., ∆*G*_E_ = *RT*ln(*k*_−4_/*k*_4_)). One major obstacle in studying kinetics of transport is that the precise measurement of kinetic parameters presents a far more daunting technical challenge than measuring thermodynamic parameters.

A free-energy landscape plot (Fig. 3) is a useful tool to visually represent the transport process, for instance whether a step is thermodynamically favorable (Zhang et al. 2015; Zhang and Han 2016). While the vertical dimension of the plot represents Gibbs free energy, the horizontal dimension can be considered as an alternative expression of the King–Altman plot. Depending on the depth (or focus) of the analysis, multiple steps in a free-energy landscape may be merged as a single one. In addition, every step in the free-energy landscape plot may be further divided into more sub-steps (see Appendix 1 for an example). In particular, each (non-diffusion limiting) step in a free-energy landscape may contain a local transition state which is related to the kinetics of the given step (see Appendix 2). Such transition states are schematically shown in Fig. 3 for both transition-1 and transition-0. A general procedure to construct a free-energy landscape plot of the two-state model, in order to comprehend relationships between functions of a uniporter and both its thermodynamic and kinetic properties, is outlined in Appendix 3.

For an influx transport process, the transport cycle runs in the clockwise direction in Fig. 2; and for an efflux transport, the cycle runs in the counter-clockwise direction. In the discussion below, we will use the kinetic parameters to name the associated “reaction” steps whenever appropriate. For example, the *k*_2_ step denotes the substrate-carrying, C_out_-to-C_in_ transition-1, and the associated free-energy barrier is denoted as $\Delta G_{2}^{{^{\ddag } }} .$ Since all steps in the transport cycle are mutually exclusive, the minimum time required by the influx (efflux) cycle is denoted as *τ*_influx,min_ (*τ*_efflux,min_).

1 $$\tau_{\hbox{influx,min} } = \tau_{1} + \tau_{2} + \tau_{3} + \tau_{4},$$

2 $$\tau_{\hbox{efflux,min}} = \tau_{-4} + \tau_{-3} + \tau_{-2} + \tau_{-1},$$

where *τ*_1_, *τ*_2_, and so on are the minimum time spent by the transporter at each step. Therefore, the maximum rate of influx transport (e.g., under so-called zero-*trans* conditions (Krupka and Deves 1981)), simply denoted as *V*_influx_, satisfies the following relationship:

3 $$\frac{1}{{V_{{\text{influx}}}}} \approx \frac{1}{{[{\text{s}}]_{{\text{L}}} k_{1}^{0} }} + \frac{1}{{k_{2} }} + \frac{1}{{k_{3} }} + \frac{1}{{k_{4} }}.$$

Similarly, the maximum rate of efflux transport, *V*_efflux_, satisfies the following relationship:

4 $$\frac{1}{{V_{{\text{efflux}}}}} \approx \frac{1}{{k_{ - 4} }} + \frac{1}{{[{\text{S}}]_{{\text{L}}} k_{ - 3}^{0} }} + \frac{1}{{k_{ - 2} }} + \frac{1}{{k_{ - 1} }}.$$

For a uniporter of molecular weight ≥50 kDa, its major conformation changes are most likely to be slower than the diffusion-dominated substrate loading and releasing. In other words, the substrate loading ($k_{1}^{0}$ and $k_{ - 3}^{0}$) and releasing (*k*_−1_ and *k*_3_) steps are usually much faster than those of the transition-0 (*k*_4_ and *k*_−4_) and transition-1 (*k*_2_ and *k*_−2_) steps, as long as the substrate concentration on the loading side is sufficiently high (≫*K*_d,L_) and that on the releasing side is low (≪*K*_d,R_, implicating relatively large *k*_−1_ and/or *k*_3_). Thus, the terms corresponding to steps $k{}_{1}^{0}$, $k_{ - 1}$, $k_{3}$, and $k_{ - 3}^{0}$ in the above equations can be omitted. In other words, depending on the experimental setup as well as properties of the transporter, the rate-limiting step(s) is very likely to be at either transition-0 or transition-1 (or sometimes both).

As an example, it has been shown that for a full cycle of glucose uptake by GLUT1, the energy barrier of the substrate-free transition-0 is higher than that of the substrate-carrying transition-1 (Lowe and Walmsley 1986). Thus, in a zero-*trans* influx (efflux) assay (i.e., under the condition of [S]_L_ ≫ *K*_M_ and [S]_R_ ≈ 0) the transport rate is dominated by the kinetic parameter, *k*_4_ (*k*_−4_), at transition-0. Therefore, the following is true:

5 $$\frac{{V_{{\text{influx}}} }}{{V_{{\text{efflux}}} }} \approx \frac{{k_{4} }}{{k_{ - 4} }} = f ( 0 ).$$

Shown in the free-energy landscape plot (Fig. 3), the above results may be interpreted in such a way that transport in the direction of a lower energy barrier at the rate-limiting step is running faster than transport in the opposite direction. This phenomenon is called asymmetric transport. The logic presented here to interpret asymmetric transport, which was also employed in earlier work by others (Lowe and Walmsley 1986), is conceptually more straight forward than a model proposed recently (Zhang and Han 2016). For GLUT1, it was estimated that at 0 °C *V*_efflux_ is more than 10 times faster than *V*_influx_ (Lowe and Walmsley 1986). In addition, the inward-facing conformation is thermodynamically favored in the absence of substrates (*f*(0) = 0.06), strongly indicating that C_in_ represents the resting state. These observations are consistent with the function of rapid glucose efflux of GLUT1, e.g., in erythrocytes delivering glucose to places of high energy demand such as the brain, though the tendency to transport asymmetrically may become less pronounced at physiological temperatures (Lowe and Walmsley 1986, Carruthers et al. 2009). Under the condition that transition-0 is the rate-limiting step, the ratio of *V*_influx_ to *V*_efflux_ is determined by ∆*G*_E_ (or *f*(0)), which in turn is influenced by interactions between the transporter and the membrane (e.g., by electric charges carried by the transporter and the electrostatic membrane potential). In general, if a uniporter is adapted to mainly transport substrates in one direction, such transporter is likely to have a value of ∆*G*_E_ compatible with such a function.

Moreover, equilibrium exchange studies on GLUT1, where influx of radiolabeled glucose was coupled with efflux of non-labeled glucose, showed that the influx rate of the radiolabeled glucose (*V*_ee_) is ~100 times faster than *V*_influx_ (i.e., in the absence of a coupled efflux) (Lowe and Walmsley 1986). Similar to Eq. 3, the following holds true for *V*_ee_:

6 $$\frac{1}{{V_{{\text{ee}}} }} \approx \frac{1}{{k_{2} }} + \frac{1}{{k_{ - 2} }}.$$

Equation 6 reflects the fact that, for equilibrium exchange, transition-0 is no longer the rate-limiting step (Fig. 2). In addition, it was estimated that *k*_2_/*k*_−2_ ≈ 10 (i.e., *f*(∞) = 0.07) (Lowe and Walmsley 1986). Thus, the rate-limiting step of the equilibrium exchange is the efflux of the non-labeled glucose, and *V*_ee_ ≈ *k*_−2_. Since the rate constant of a reaction step is reciprocally related to the forward energy barrier of its local transition state by the Arrhenius equation (Appendix 2), the *V*_ee_/*V*_influx_ (≈*k*_−2_/*k*_4_) ratio of 100 suggests that the energy barrier of substrate-carrying transition-1 (in particular ∆*G*_−2_^‡^) is ~5 *RT* (i.e., *RT*ln(100)) lower than that of substrate-free transition-0 (∆*G*_4_^‡^). This point will be discussed further in the last section.

*Trans*-acceleration is a phenomenon that uptake of radiolabeled substrate is enhanced by the existence of (other types of) non-radiolabeled substrates at the opposite side of the membrane. While GLUT1 and GLUT3 display characteristics of *trans*-acceleration, GLUT4 and GLUT2 lack such trait (Nishimura et al. 1993). Similar to the above discussion, *trans*-acceleration in GLUT1 can be explained by the fact that *k*_−2_ ≫ *k*_4_, given the argument that, once the second substrate is added on the *trans*-side, the rate-limiting step switches from the *k*_4_ step to the *k*_−2_ step in the King–Altman plot. In contrast, absence of *trans*-acceleration suggests that GLUT4 has an energy barrier for the substrate-carrying transition-1 (the *k*_−2_ step) comparable with transition-0 (the *k*_4_ step), such that the *V*_ee_/*V*_influx_ (≈*k*_−2_/*k*_4_) ratio becomes close to 1 (assuming the remaining profile of thermodynamic parameters of GLUT4 are the same as that of GLUT1). Consistent with its *trans*-acceleration property, GLUT1 also shows asymmetry in zero-*trans* influx/efflux assays as mentioned above; in contrast, GLUT4 displays kinetic symmetry (Taylor and Holman 1981). In particular, for GLUT1, the rate-limiting steps for zero-*trans* influx and efflux are *k*_4_ and *k*_−4_, respectively, and thus *V*_influx_/*V*_efflux_ (≈*k*_4_/*k*_−4_, Eq. 5) equals to ~1/10, indicating asymmetry. In contrast, because of equal heights for both transition-0 and -1 in GLUT4, its ratio of *V*_influx_/*V*_efflux_ (≈*k*_4_/*k*_−2_) becomes close to 1, indicating symmetry. Interestingly, studies with chimeric constructs showed that transmembrane helix 6 (TM6) of GLUT4 is responsible for a lowering of the energy barrier at transition-0 compared with that of GLUT1 (Vollers and Carruthers 2012). It is noted that an MFS transporter contains two domains, N- and C-domain, which are related by a pseudo two-fold symmetry (Fig. 1). TM6 is located on the surface of the N-domain, directly contacting with the lipid bilayer. However, it is not involved in the inter-domain interface where substrates are bound and conformational changes occur. As a certain degree of intra-domain flexibility is required by the function of an MFS transporter (Quistgaard et al. 2016), in GLUT4 the interface between TM6 and other TM helices within the N-domain may be more frictionless, rendering the transition-0 state more flexible and thus less strained during the conformational change.

Therefore, both its thermodynamic and kinetic properties are essential for proper functioning of a uniporter. It is important to understand how external free energy, including electrochemical potential of the substrate, drives the thermodynamic process of the transporter, and how the substrate binding affects the kinetic property of the transporter. The simple, two-state, four-step model described here should provide meaningful interpretations in both aspects, at least qualitatively.

## Reduction of the energy barrier of the transition state

On the cell surface, various potential substrates/ligands may exist in the surroundings of a uniporter, and they compete for the uniporter or cooperate with each other for utilizing the transporter. For instance, both glucose and lactate (a product of glycolysis) compete for transport by GLUT1 (Simpson et al. 2007). In general, these substances may be divided into three classes: those (1) whose binding increases the transition rate relative to transition-0; (2) whose binding has no effect on the transition rate; and (3) whose binding reduces the transition rate. Borrowing terminology from receptor-mediated signaling (Zhang et al. 2016), in terms of promoting the conformational transition of the uniporter, these three types of ligands may be considered as agonists, antagonists, and (partial) inverse agonists. While glucose transported by GLUT1 belongs to the first type of ligands, glucose transported by GLUT4 seems to belong to the second (Nishimura et al. 1993). In addition, aspartic acid (and Na^+^) transported by Glt_Ph_ seems to belong to the third type (Akyuz et al. 2015). Clearly, not all ligands of the third type are necessarily inhibitors. In addition, it is hypothetically feasible to imagine another type of inhibitors, namely one that over-stabilizes a transition state in a manner that the transition state simply becomes a deep energy valley, mimicking a classical transition-state analog inhibitor that traps the enzyme at the transition state (sometimes by forming a covalent bond). The affinity of such an inhibitor must be so strong that it would over-compensate ∆*G* ^‡^. Nevertheless, such inhibitors for transporters remain to be discovered. Therefore, whether a given ligand is a good substrate for a transporter may not only depend on its affinity strength in the loading step, but also on how it affects the energy barrier of the transition state.

Proper functioning of a uniporter depends on the balance between efficient transport and prevention of potential leakage of non-specific ligands. Substrate binding-induced reduction of the energy barrier results in such a balance, thus making biological sense (Klingenberg 2007). This is especially true for those transporters that are more or less constitutively expressed at the cell surface (such as GLUT1). It may be of less importance, however, for transporters that are dynamically regulated by other mechanisms, for example for insulin-induced cell surface expression of GLUT4 (James et al. 1989). On the one hand, transition-0 is more likely to be the rate-limiting step, so that the transporter would not switch freely between the C_out_ and C_in_ states, thus limiting incidental leakage. On the other hand, in order for the transport cycle to proceed, the energy barrier of transition-0 (which is part of the transport cycle of the uniporter) must be reasonably low, in order to render the barrier accessible to thermal motion. An “ideal” substrate of a given uniporter could be defined as a ligand that decreases the energy barrier of transition-1 relative to transition-0, thus (1) increasing the rate of conformational transition as well as the transport cycle and (2) competing more effectively with other potential substances in utilizing the transporter.

Hypothetically, there may be numerous ways for a substrate to affect the energy barrier (∆*G* ^‡^). Lowering ∆*G* ^‡^ of a uniporter does not consume extra energy input, including the chemical potential of the substrate. Instead, substrate binding per se plays a role in reducing ∆*G* ^‡^. The substrate binding-induced reduction of ∆*G* ^‡^ was termed as intrinsic binding energy (Klingenberg 2006). We would like to emphasize that this “driving” energy is gained from substrate binding in the first half of the transition but is immediately released in the second half of the same transition. Since the transition rate is mainly determined by the forward kinetic rate, the overall effect of the ∆*G* ^‡^ reduction at the rate-limiting step is acceleration of transport. It is well known that substrate binding may induce so-called occluded conformations, which have been captured in a number of reported crystal structures (Deng et al. 2015). Thus, the occluded conformations are likely to have higher affinity towards substrates than the C_in_ and C_out_ states (Quistgaard et al. 2016), at least under the in vitro conditions. In lipid bilayers where both mechanical membrane tension and electrostatic membrane potential may be present, such an occluded conformation may or may not be thermodynamically stable. However, as long as it is not over-stabilized relatively to the following substrate-releasing state, an occluded state would not prevent proceeding of the transport. The two-state model remains valid should the transient occluded state be merged with neighboring sub-steps of the transition. Furthermore, the transition-state functions as a mechanism for strong substrate selectivity. Analogously, stabilization of the transition state of an enzyme–substrate complex is a common mechanism in enzyme catalysis as well as selectivity. For transporters, such a mechanism has been specifically termed as induced transition fit (Klingenberg 2007). It should be stressed that reduction of ∆*G* ^‡^ is not driven by the chemical potential of the substrate, because during the transition-1 the substrate has already bound to the transporter thus being irrelevant to the external concentration(s) of the substrate. Detailed structure studies of uniporters may provide information on the mechanism of substrate binding-mediated reduction of ∆*G* ^‡^, as exemplified in mechanistic discussion on GLUT1 crystal structure (Deng et al. 2014). Similar mechanisms may also exist for secondary active transporters, for example for members of the MFS family (Quistgaard et al. 2016), where external energy provides an additional driving force to overcome ∆*G* ^‡^ (Zhang et al. 2015).

Using the free-energy landscape plot (Fig. 3) as a tool, we will discuss the substrate binding-induced reduction of the energy barrier of the transition-1 relative to transition-0 in more detail. Let’s first compare C_out_-to-C_in_ transitions with and without a bound substrate. The substrate binding-induced reduction of the energy barrier is denoted as $\Delta \Delta G^{\ddag }_{\text{OI}} \equiv \Delta G^{\ddag }_{ 2} - \Delta G^{\ddag }_{ - 4}$. A negative value of $\Delta \Delta G^{\ddag }_{\text{OI}}$ would indicate that *k*_2_ > *k*_−4_. In general, the three above-mentioned ligand types correspond to the three situations whereby $\Delta \Delta G^{\ddag }_{\text{OI}}$ is either smaller, equal to, or larger than zero. The energy reduction may also be presented as *RT*ln(*K*_d,T_/*K*_d,out_). Here, *K*_d,T_ is a hypothetical dissociation constant at the transition state, which is an intrinsic property of the transporter for a given substrate. An “ideal” substrate would have a *K*_d,T_ value smaller than that of *K*_d,out_, thus lowering the energy barrier of the transition state by $\left| {\Delta \Delta G^{\ddag }_{\text{OI}} } \right|$. Consequently, the transition rate of the *k*_2_ step would increase by a factor of *K*_d,out_/*K*_d,T_ relative to that of the *k*_−4_ step. The difference between *K*_d,T_ and *K*_d,out_ is likely to be contributed mainly by the corresponding enthalpy difference (Appendix 1), for example a variation of the number of hydrogen bonds induced by substrate binding. Similarly, for a C_in_-to-C_out_ transition the reduction is denoted as $\Delta \Delta G^{\ddag }_{\text{IO}}$ $(\equiv  \Delta G^{\ddag }_{-2}- \Delta G^{\ddag}_{4})$ and equals to *RT*ln(*K*_d,T_/*K*_d,in_). A negative value of $\Delta \Delta G^{\ddag }_{\text{IO}}$ would indicate that *k*_−2_ > *k*_4_. Therefore, $\Delta \Delta G^{\ddag }_{\text{OI}}$ does not necessarily equate to $\Delta \Delta G^{\ddag }_{\text{IO}}$, and their difference $(\Delta \Delta G^{\ddag }_{\text{OI}} - \Delta \Delta G^{\ddag }_{\text{IO}} )$ is exactly the same as the differential binding energy, ∆*G*_D_. For instance, for a transporter of negative ∆*G*_D_, the substrate binding-induced reduction of the energy barrier in the efflux direction would be less significant than that in the influx direction.

Given the picture presented here on their thermodynamics and kinetics, structural studies on uniporters should focus on understanding how the 3D structures implement the energy landscape suitable for efficient transport. To improve our understanding of the structure–function relationship in uniporters, especially in relation to the function of substrate binding and energy coupling, a number of questions require urgent attention: What is the structural basis of substrate specificity and affinity (*K*_d_)? How does substrate binding lower the energy barrier (∆*G* ^‡^) of transition-1 relative to transition-0? How may a regulatory ligand (or mutation) affect the free-energy landscape including energy barriers of the transition states? Finding answers to these important questions should enable critical rethinking of the role of substrate binding for transporter function.

## Figures and Tables

**Fig. 4:**
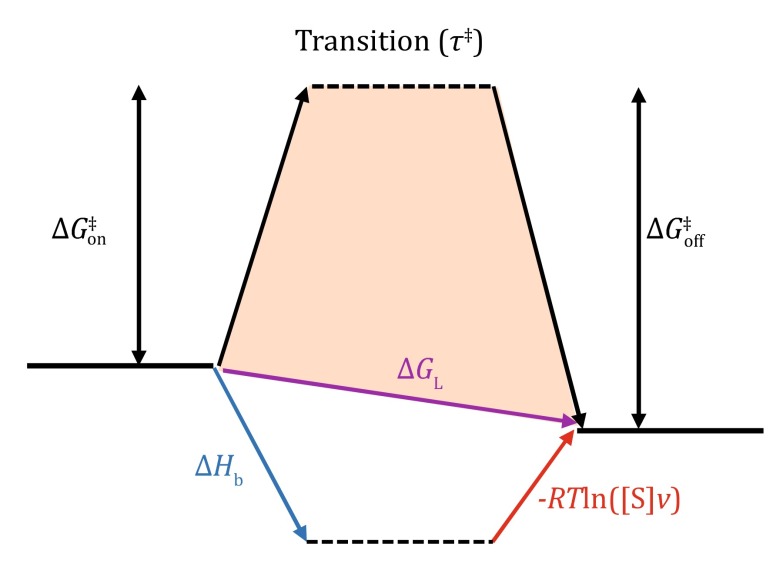
Dissection of the substrate binding step

**Fig. 5:**
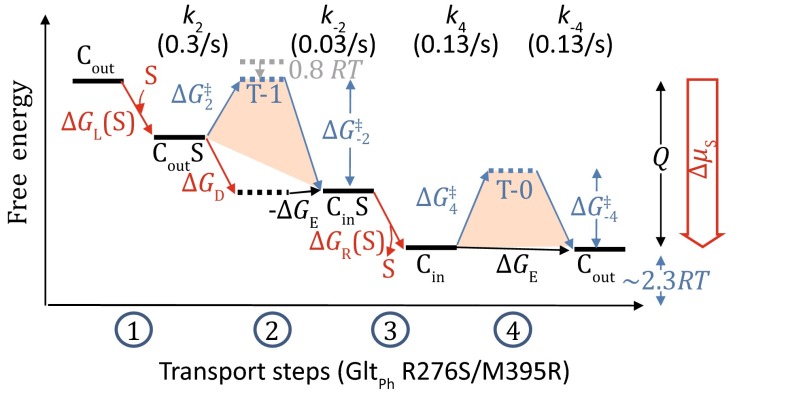
Free energy landscape of transport steps (Glt_Ph_ R276S/M395R)

## Appendix

### Appendix 1: Dissociation constant

If the dissociation constant *K*_d_ is determined experimentally, the substrate binding may be represented as one step in the energy landscape plot. Instead, if both the on- and off-rate constants, *k*_on_ and *k*_off_, are determined, the kinetics of the above binding step may be modeled as forward and backward steps separated by a “local” transition state (Fig. 4). Such a local transition state may have relatively high forward and backward energy barriers ($\Delta G^{\ddag }_{\text{on}}$ and $\Delta G^{\ddag }_{\text{off}}$), associated with the on and off rates of the “reaction” step. By definition, a transition state is hard to be captured experimentally, because its life time (τ^‡^) is short for the technique being used.

7 $$K_{\text{d}} \equiv k_{\text{off}} /k_{\text{on}} = { \exp }(\Delta H_{\text{b}} )/v,$$

where ∆*H*_b_ (<0) is the binding enthalpy and *v* can be considered as the specific volume occupied by the substrate in the binding site (if the degrees of rotation freedom can be ignored);

8 $$\Delta G_{\text{L}} \equiv - RT{ \ln }\left( {\left[ {\text{S}} \right]/K_{\text{d}} } \right) = \Delta H_{\text{b}} - T\Delta S_{\text{b}} = \Delta G^{\ddag }_{\text{on}} - \Delta G^{\ddag }_{\text{off}},$$

where ∆*S*_b_ (≡*R*ln([S]*v*)) is the entropy change during the binding;

9 $$\Delta G^{\ddag }_{\text{on}} = - RT{ \ln }(\tau^{\ddag } k_{\text{on}} [{\text{S}}]) = \Delta G^{\ddag }_{ 0} - T\Delta S_{\text{b}},$$

where $\Delta G^{\ddag }_{ 0} ( \equiv - RT{ \ln }(\tau^{\ddag } k_{\text{on}} /\nu ))$ is independent of [S]. In kinetic terms, 1/(*k*_on_[S]) may be considered as the life time of “ground” state before jumping to the transition state which has a life time of *τ* ^‡^ (Note that $\Delta G^{\ddag }_{ 0}$ and *τ* ^‡^ are inter-dependent)

10 $$\Delta G^{\ddag }_{\text{off}} = - RT{ \ln }(\tau^{\ddag } k_{\text{off}} ) = \Delta G^{\ddag }_{ 0} - \Delta H_{\text{b}}.$$

∆*G*_L_ contains two terms, namely the entropy contribution −*RT*ln([S]*v*) and enthalpy (∆*H*_b_). In most biochemical assays, it holds true that [S] ≪ 1/*v*. Thus, the entropy change is negative during binding, and its contribution to the binding free energy is thermodynamically unfavorable. As [S] increases, ∆*G*_L_ decreases (becoming less unfavorable). When [S] reaches *K*_d_, ∆*G*_L_ becomes zero. When [S] approaches to 1/*v*, ∆*G*_L_ is dominated by the enthalpy term.

### Appendix 2: Arrhenius equation

For non-diffusion-limiting reaction, for example the transition-0 in the two-state model (Fig. 1), the following Arrhenius equations hold true.

11 $$k_{ 4} = { \exp }( - \Delta G^{\ddag }_{ 4} /RT)/\tau^{\ddag }_{ 4},$$

12 $$k_{ - 4} = { \exp }( - \Delta G^{\ddag }_{ - 4} /RT)/\tau^{\ddag }_{ 4}.$$

Here, 1/*k*_4_ and 1/*k*_−4_ can be considered as the life time of empty C_in_ and C_out_ states, respectively. Note that $\tau^{\ddag }_{ 4}$ and $\Delta G^{\ddag }_{ 4}$ are coupled, and thus cannot be determined independently.

Microscopic activation rates, *k*_4_ and *k*_−4_, are independent of substrate concentration. However, macroscopic rates, such as *V*_influx_ and *V*_efflux_, follow the Michaelis–Menten kinetics. When the transporter is not fully occupied by substrate, all rates of first-order reaction steps should be modified by a factor of [S]/([S] + *K*_d_). From Eq. 3, we have a more accurate equation for *V*_influx_

13 $$\begin{aligned}                                                                                                                                                                                                                                                                                                              
\frac{1}{V_{\text{influx}}}&=\frac{1}{[{\text{S}}]k_{1}^{0}}+\left(\frac{1}{k_{2}}+\frac{1}{k_{3}}+\frac{1}{k_{4}}\right)\                                                                                                                                                                                                     
\left(\frac{[{\text{S}}]+k_{\text{d,out}}}{[{\text{S}}]}\right)\\&=\frac{1}{k^{\prime}}\left(\frac{k^{\prime}}{[{\text{S}}]k_{1}^{0}}\right)+\frac{1}{k^{\prime}}\left(\frac{[{\text{S}}]+K_{\text{d,out}}}{[{\text{S}}]}\right)\\&=\frac{1}{k^{\prime}}\left(1+\frac{K_{\text{M,influx}}}{[{\text{S}}]}\right),\end{aligned}$$

where $$\frac{1}{k^{\prime}} \equiv \frac{1}{k_{2}} + \frac{1}{k_{3}} + \frac{1}{k_{4}} , \,{\text{and}} \ K_{{{\text{M}}},{\text{influx}}} \equiv \frac{k_{ - 1} +
k^{\prime}}{k_{1}^{0}} ,$$

14 $$\therefore \quad V_{\text{influx}} = k '\frac{{[{\text{S}}\}_{L} }}{{[{\text{S}}]_{L} + K_{{{\text{M}},{\text{influx}}}} }}.$$

Under the conditions of zero-*trans* influx (i.e., [S]_R_ ≈ 0 and [S]_L_ ≫ *K*_M,influx_), *V*_influx_ equals to *k*’ (≈*k*_4_, assuming *k*_2_, *k*_3_ ≫ *k*_4_). In addition, the formula of *K*_M_ may partially explain a common observation on transporters, namely that an increase in transport rate (*k*’) is often accompanied by a reduction in affinity (i.e., increase in *K*_M_), the so-called Haldane relationship (Lowe and Walmsley 1986).

### Appendix 3: General strategy to construct the free-energy landscape plot for a uniporter

The free-energy landscape plot is a useful tool to comprehend relationships between functions of a uniporter and both its thermodynamic and kinetic properties. A general procedure to construct such a plot from experimental data is outlined below.

(i) Use techniques such as smFRET to measure the partition function *f*([S]) of the transporter between the two conformations (C_in_ and C_out_), including its values under the extreme conditions *f*(0) and *f*(∞). These data should allow determination of other thermodynamic parameters ∆*G*_D_, ∆*G*_E_, *K*_d,in_, *K*_d,out_, and *K*_d,app_ (Zhang et al. 2015). In case that *f*(0) = *f*(∞), both *K*_d,in_ and *K*_d,out_ equal to *K*_d,app_ which can be measured with more traditional techniques such as SPR or ITC. It should be noted that *f*([S]) measured under a non-membrane condition may differ from that in the presence of membrane tension and membrane potential. This is especially true when the transporter and/or substrate carry electric charges. Ideally, *f*([S]) should be measured under conditions mimicking the real cellular membrane.
(ii) Use kinetic methods to determine *V*_ee_, *V*_influx_, *V*_efflux_, *K*_M,influx_, and *K*_M,efflux_, as functions of temperature (Lowe and Walmsley 1986). Determine whether transition-1 or transition-0 is the rate-limiting step. For example, if the rate of equilibrium exchange (*V*_ee_) is significantly faster than the rate of influx transport (*V*_influx_), the transition-0 is likely to be the rate-limiting step in the influx transport. Transition rate may also be estimated from dwelling time (*τ*) of smFRET assays, e.g., in the absence and presence of saturated substrate. The values of 1/*τ* represent the transition rates (See Appendix 1 for an example).
(iii) Further, use the van’t Hoff-Arrhenius plot (i.e., ln(*V*_ee_), ln(*V*_influx_), or ln(*V*_efflux_) vs 1/*T*) to estimate the corresponding transition-state energy barriers (∆*G* ^‡^), in particular the enthalpy terms (Appendix 5).
(iv) Construct the free-energy landscape plot of the uniporter using parameters obtained from the steps described above.

### Appendix 4: Glt_Ph_ transporter

The following discussion serves as an example of how to use experimental data to build a two-state model (Fig. 5). It is based on smFRET data from Akyuz et al.(2015). We will discuss the R276S/M395R mutant of Glt_Ph_, because the WT Glt_Ph_ has some bizarre behaviors (see below).

(i) In the absence of substrate but the presence of detergent, the ratio of C_out_ and C_in_ population is close to 1:1. Thus ∆*G*_E_ ≈ 0.
(ii) In the presence of both substrates and detergent, C_in_ state becomes dominant. Assuming that the partition function *f*(∞) < 1/10, one may estimate that ∆*G*_D_ < −2.3 *RT*.
(iii) In proteoliposomes, the average time of a transition “cycle” in the absence of substrate (*τ*_free_) is 15 s (=2/(0.13 s^−1^)), where the factor 2 comes from the two FRET signals in each cycle. In the presence of substrate gradient, the observed average time of a transition “cycle” (*τ*_obs_) is 20 s (=2/(0.1 s^−1^)), and the average time of a substrate uptake cycle (*τ*_influx_) is 30 s (=1/(0.03 s^−1^)). Assume that *α* fraction of transporters bind with substrates.
  15 $$\tau_{\text{obs}} = \alpha \tau_{\text{influx}} + ( 1- \alpha )\tau_{\text{free}},$$
  16 $$\therefore \quad \alpha = \left( {\tau_{\text{obs}} - \tau_{\text{free}} } \right)/\left( {\tau_{\text{influx}} - {\tau }_{\text{free}} } \right) = 1/3,$$
  17 $$[{\text{S}}]/K_{\text{d,out}} = \alpha /( 1- \alpha ) = 1/2.$$
  Thus, the transporters were not saturated under the experimental condition.
(iv) The observed uptake rate (*V*_influx_) of substrate (radiolabeled aspartic acid) is 0.03/s; and the transition rate (*k*_4_ ≈ *k*_−4_) in the absence of substrate is 0.13/s. Because only 1/3 of transporters are occupied by substrates on the loading side, according to Eq. 13, both the rates *k*_2_ and *k*_4_ should be modified by a factor of 1/3 (see Eq. 14).
  18 $$ V_{\text{influx}}^{ - 1} \approx 3\left( {(k_{2} )^{ - 1} + (k_{4} )^{ - 1} } \right),$$
  $$\therefore \quad k_{2} \approx 0.3/{\text{s}}.$$

Thus, the rate at transition-1 is slightly faster than that at transition-0, and the ratio of *k*_2_ to *k*_4_ (≈*k*_−4_) is ~2.3. It indicates that the $\Delta G^{\ddag }_{2}$ barrier is marginally lower (by 0.8 *RT*) than the $\Delta G^{\ddag }_{ - 4}$ barrier. In contrast, because ∆*G*_D_ < −2.3 *RT*, the $\Delta G^{\ddag }_{ - 2}$ barrier is higher (by > 1.5 *RT*) than the $\Delta G^{\ddag }_{ 4}$ barrier.

Note that the behaviors of the WT Glt_Ph_ are significantly different from the R276S/M395R mutant. In particular, for WT the rate at transition-1 is 10 times slower than that at transition-0 state, indicating a 2.3 *RT* increase at transition-1 barrier compared to transition-0. Further, it suggests that one effect of the mutation (R276S/M395R) is to reduce the free-energy barrier of the transition-1 relative to WT.

### Appendix 5: van’t Hoff equation

Arrhenius equation on the relationship between transition rate *V* and activation energy ∆*G* ^‡^ (≡∆*H* ^‡^ − *T*∆*S* ^‡^) is the following:

19 $$V = \exp \left( { - \Delta G^{\ddag } /RT} \right)/\tau^{\ddag }.$$

Assuming that *τ* ^‡^, ∆*H* ^‡^, and ∆*S* ^‡^ are independent of temperature *T*, the van’t Hoff equation on the relationship between enthalpy ∆*H* ^‡^ and variation of *V* as temperature changes is the following:

20 $$\Delta H^{\ddag } = \Delta (\ln V)RT^{2} /\Delta T.$$

Since the transition state is usually more rigid than the “ground” state, the corresponding entropy term may further increase the transition-state energy barrier. An example of applying this method is shown in Lowe and Walmsley (1986). However, the estimations of ∆*G* ^‡^ (∆*H* ^‡^) values shown there, for the energy barriers at the *k*_4_, *k*_−4_, *k*_−2_, and *k*_2_ steps, were 173, 127, 88, and 32 kJ/mol (or 72, 53, 37, and 13 times of *RT*), respectively. They are obviously too high for activation energy of a uniporter which does not have external energy input except substrate chemical potential. One probable reason for this over-estimation is that the assumption for the van’t Hoff equation (that activation enthalpy is independent of temperature) might not hold true for some membrane proteins. This problem may be particularly serious at low temperature, because of significant change of the flexibility of lipid bilayer (and even physical phase of lipids) with temperature. Another explanation may be that the experimentally determined *V*_influx_ and *V*_efflux_ (under zero-*trans* condition) also include other terms in addition to the rate constant of the rate-limiting step such that Eq. 19 was not true.

## Footnotes

1 Originally, the subscript “E” in ∆*G*_E_ depicted “elastic” conformational energy stored in the non-resting state (Zhang et al. 2015). However, since this energy does not follow Hook’s law, it is not elastic in nature. Thus, the subscript “E” better refers to “empty”, i.e., unloaded states of the transporter.
